# Ethoxyquin: An Antioxidant Used in Animal Feed

**DOI:** 10.1155/2013/585931

**Published:** 2013-04-30

**Authors:** Alina Błaszczyk, Aleksandra Augustyniak, Janusz Skolimowski

**Affiliations:** ^1^Department of General Genetics, Molecular Biology and Plant Biotechnology, Faculty of Biology and Environmental Protection, University of Łódź, Banacha 12/16, 90-237 Łódź, Poland; ^2^Department of Organic Chemistry, Faculty of Chemistry, University of Łódź, Tamka 12, 91-403 Łódź, Poland

## Abstract

Ethoxyquin (EQ, 6-ethoxy-1,2-dihydro-2,2,4-trimethylquinoline) is widely used in animal feed in order to protect it against lipid peroxidation. EQ cannot be used in any food for human consumption (except spices, e.g., chili), but it can pass from feed to farmed fish, poultry, and eggs, so human beings can be exposed to this antioxidant. The manufacturer Monsanto Company (USA) performed a series of tests on ethoxyquin which showed its safety. Nevertheless, some harmful effects in animals and people occupationally exposed to it were observed in 1980's which resulted in the new studies undertaken to reevaluate its toxicity. Here, we present the characteristics of the compound and results of the research, concerning, for example, products of its metabolism and oxidation or searching for new antioxidants on the EQ backbone.

## 1. Introduction

During storage of animal feed many different processes may occur which alter their initial natural proprieties. First of all, lipids undergo peroxidation, the process during which they are deteriorated in a free radical autocatalytic oxidation chain reaction with atmospheric oxygen. Lipid autooxidation is a cascade phenomenon ensuring continuous delivery of free radicals, which initiate continuous peroxidation. This process results in food rancidity which manifests itself as the change of taste, scent, and color as well as decrease in shelf life of the product. Natural or synthetic antioxidants are usually used to slow down or stop lipid peroxidation and in consequence to preserve freshness of the product. Many natural antioxidants, such as tocopherols, vitamin C, flavonoids, for a short period, may be effective in food preserving, but in many cases such protection is not sufficient. Therefore synthetic antioxidants are widely used, among which BHT (butylated hydroxytoluene), BHA (butylated hydroxyanisole), and EQ (ethoxyquin) are the most frequent. However, some effects of synthetic antioxidants are not always beneficial for our health. Antioxidants such as BHA or BHT have been widely used for many years to preserve freshness, flavor, and colour of foods and animal feeds as well as to improve the stability of pharmaceuticals and cosmetics. There are many controversies about the use of these two antioxidants in foods. Some experimental studies have reported that both BHT and BHA have tumour-promoting activity [1, 2]. On the other hand, there were reports on anticarcinogenic properties of these antioxidants when they are used at low concentrations [3]. Human exposures are at least 1000-fold below those associated with any neoplastic actions in laboratory animals thus it is assumed that they are not harmful for human beings [3, 4]. 

 The third compound, EQ, is one of the best known feed antioxidants for domestic animal and fish. Its unquestionable advantage is its high antioxidant capacity and low production costs. However, some of the authors have suggested that it is responsible for a wide range of health-related problems in dogs as well as in humans [5–9]. Due to the increased use of this antioxidant it was nominated by FDA (US Food and Drug Administration) for carcinogenicity testing [10]. The tests were carried out by Monsanto Company (USA), EQ producer, and after that in 1977 FDA requested for optional lowering of the maximum level of EQ in complete dog foods from allowed 150 ppm (0.015%) to 75 ppm (0.0075%). At the same time new studies were started by the Pet Food Institute to determine whether even lower EQ levels (between 30 and 60 ppm) would provide antioxidant protection for dog food [11]. 

 Ethoxyquin is also known as Santoquin, Santoflex, Quinol. It was originally developed in rubber industry to prevent rubber from cracking due to oxidation of isoprene [12]. The Monsanto Company (USA) taking into account its high antioxidant efficiency and stability as well as low costs of synthesis refined it later for use as a preservative in animal feeds because it protects against lipid peroxidation and stabilizes fat soluble vitamins (A, E). Presently, ethoxyquin is used primarily as an antioxidant in canned pet food and in feed intended for farmed fish or poultry.

The use of ethoxyquin is not permitted in foods intended for human, except preserving powdered paprika and chili colour and using it as an antiscald agent in pears and apples (inhibition of “brown spots” development). However, because EQ is used as a feed antioxidant it can be also found in other products intended for human consumption like fish meal, fish oils, and other oils, fats, and meat (Table 1). An acceptable daily intake (ADI) of EQ for human (0–0.005 mg kg^−1^ bw) based on the results obtained from studies on dogs was established in 1998 [13, 14].

 This paper presents characteristics of ethoxyquin with regard to its properties, metabolism, toxicity, possible carcinogenicity, and antioxidant activity.

## 2. Physical and Chemical Properties of Ethoxyquin (EQ)

**Figure 1:**
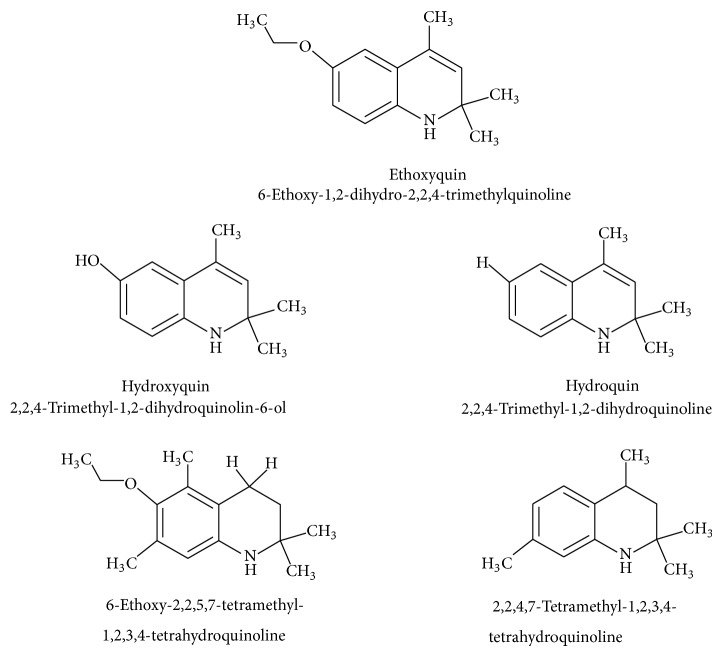
Chemical structure of ethoxyquin (EQ) and of some new compounds synthesized on ethoxyquin backbone with promising antioxidant properties (according to de Koning [12], Dorey et al. [15] and Błaszczyk and Skolimowski [17]).

For the first time EQ was synthesized in 1921 by Knoevenagel [15]. The synthesis was based on condensation of aniline with molecules of acetone or its analogues [15, 16]. Błaszczyk et al. [16] synthesized EQ from p-phenetidine (4-ethoxyaniline) and diacetone alcohol in the presence of p-toluenesulfonic acid or iodine. Pure ethoxyquin (EQ; 6-ethoxy-1,2-dihydro-2,2,4-trimethylquinoline; CAS number 91-53-2; Figure 1) is a light yellow liquid, but it changes color to brown if it is exposed to oxygen [12]. It also tends to polymerize on exposure to light and air. The scent of EQ is described as mercaptan like. As a nonpolar substance EQ is soluble only in organic solvents. Some of the additional properties of EQ are presented in Table 2.

## 3. Biotransformation of EQ

### 3.1. Animals

Ethoxyquin is rapidly absorbed from gastrointestinal tract of laboratory animals like rats and mice. Peak blood concentration of the compound is observed within 1 h. Distribution of EQ in animal body is similar when it is administered orally and intravenously. Small amounts of parent EQ were detected in liver, kidney, and adipose tissue and fish muscles [20–24]. It is excreted predominantly as metabolites via urine. Metabolism of EQ was studied in rats, mice, dogs, chickens, and fish, as well as in plants [13, 21, 25]. It is not fully described but some metabolites were identified (Table 3). The most important EQ metabolites observed in rat urine and bile result from O-deethylation at position 6-C, and then conjugation with sulphate or glucuronide residues. The other metabolic pathways include hydroxylation and glucuronidation at position 8-C, deethylation at 6-C and epoxidation between positions 3-C and 4-C [21]. The main metabolites observed may be different depending on animal species. In mice mainly glucuronide metabolites were detected while in rats those result from conjugation of EQ with sulfate.

 In the studies of Bohne et al. [23, 26] parent EQ, dem-ethylated EQ (DEQ), quinone imine (QI), and EQ dimer (EQDM) were observed in salmonid fish after long-term dietary exposure to EQ. It was in agreement with the results obtained earlier by Skaare and Roald [27]. EQ is considered as a model inducer of phase II enzymes involved in the metabolism of xenobiotics, but influence of EQ on phase I enzyme gene transcript levels was also observed [28, 29]. The key role in mediating phase I reactions (e.g., oxidation or reduction) producing more hydrophilic compounds is played by the CYP (cytochrome P450) enzyme family. Bohne et al. [28, 29] observed the alteration of CYP3A gene expression; an increase in the amount of CYP3A transcripts was detected in salmon after feeding them with the diet containing EQ at the highest dose used (1800 mg kg^−1^). The authors speculate that EQ may regulate CYP3 gene expression by interaction, for example, with pregnane X nuclear receptor (PXR) whose function is to sense the presence of toxic xenobiotics and in response enhance the expression of proteins involved in their detoxification. On the other hand, CYP1A1 gene expression, which was described as an exposure biomarker to both endogenous and exogenous compounds [30], was not increased after dietary exposure of salmonid fish to EQ and during the depuration period a trend toward downregulation was noted [28, 29]. Such an effect was observed despite the increase in the expression of AhR mRNA (AhR, cytosolic transcription factor responsible for changes in gene transcription). This effect can be explained in several ways. For example, the parent EQ may bind CYP1A1 protein and as a result may inhibit the gene expression and activity of protein [31]. Hepatic antioxidant response elements (ARE) or AhR repressor (AhRR) together with basic-helix-loop-helix-PAS (Per-AhR/ARNT-Sim homology sequence) of transcription factor usually associated with each other to form heterodimers (AhR/ARNT or AhRR/ARNT) may be also involved in the CYP1A1 downregulation process. These heterodimers can influence gene expression by binding ARE sequences in the gene promoter regions [32].

 However, EQ as other phenolic antioxidants, first of all causes induction of phase II xenobiotic-metabolizing enzymes. Bohne et al. [28, 29] observed elevated dose-related uridine diphosphate glucuronosyl-transferase (UDPGT) mRNA expression after dietary exposure to EQ. As UDPGT reacts with the compounds that have the hydroxyl group (-OH) parent EQ cannot be the potential substrate for glucuronidation, only its metabolites, for example DEQ (6-hydroxy-2,2,4-trimethyl-1,2-dihydroquinoline; Table 3), the metabolite identified by Berdikova Bohne et al. [26] in Atlantic salmon. Changes in the expression of glutathione S-transferase (GST) gene were also observed after feeding animals with EQ containing feed. The alterations in GST activity caused by EQ were documented in Atlantic salmon [28, 29], in rodents [33, 34], and in nonhuman primates [35]. In addition to UDPGT and GST, some other enzymes are involved in phase II metabolism of EQ, for example, NADP(H) : quinone oxidoreductase and epoxide hydrolase [36].

 The expression pattern of both phase I and II enzymes involved in EQ metabolism may vary in different animals and should be considered in relation to the ratio of parent EQ and its metabolites (first of all DEQ, QI, and EQDM) in the liver [29, 37]. The research concerning this issue is currently in progress.

### 3.2. Plants

 Ethoxyquin is also registered as an antioxidant to control scald (browning) in apples and pears. The EQ plant metabolites/degradation products were detected, and it was shown that in general they are different from those observed in animals (Table 3). In pears treated with ring-labeled [^14^C]ethoxyquin the following compounds were detected: N–N and C–N dimers, demethylethoxyquin (DMEQ), dehydrodemethylethoxyquin (DHMEQ), and dihydroethoxyquin (DHEQ) [14, 25]. It was shown that ethoxyquin was rapidly degradated or metabolized but itself it was not translocated into the pulp of fruit where the residues were detected (less than 0.5% of total radioactive residue was EQ). Toxicity of EQ metabolites, MEQ, DHMEQ, and DHEQ was studied in dogs (oral administration, single doses of 50 to 200 mg kg^−1^ bw), and it was found that they did not show any significant toxicity. In the report of Gupta and Boobis [14] the rank order of toxic potency for the plant metabolites and EQ is MEQ < EQ < DHEQ < DHMEQ (the least toxic first). MEQ, DHMEQ, and DHEQ were also evaluated for genotoxicity in *in vitro* and *in vivo* tests. The compounds did not cause gene mutations in *Salmonella typhimurium* and *Escherichia coli* strains, but they induced chromosomal aberrations or/and endoreduplication in Chinese hamster ovary cells. On the other hand, plant metabolites/degradation products did not exhibit genotoxic potential *in vivo*. ADI intake for humans for MEQ, DHMEQ, and DHEQ was estimated at the same level as for EQ (0–0.005 mg kg^−1^ bw).

## 4. Antioxidant Activity of EQ

 EQ possesses high-antioxidative activity. It is very efficient in protecting lipids which are present in food against oxidization [38, 39]. Specifically it is used to retard oxidation of carotene, xanthophylls, and vitamins (like vitamins A or E). In animals treated with ethoxyquin three times higher level of vitamins A and E in blood plasma was observed [40]. This finding suggests that an organism is using EQ instead of natural antioxidants. High efficiency of this antioxidant results not only from chemical features of EQ itself but also from the fact that products of its oxidation also possess antioxidative properties [12, 39, 41].

 Studies on EQ antioxidant properties were performed by Taimr [39] with the use of alkylperoxyls, and it was shown that the reaction rate of EQ with them is very high. In the presence of high oxygen concentrations EQ reacts with alkylperoxyl molecule to form aminyl radical (6-ethoxy-2,2,4-trimethyl-1,2-dihydroquinolin-1-yl) which subsequently may enter various pathways. In nonoxidizing conditions it can be stabilized both by the loss of methyl group and aromatization of heterocycle to form 2,4-dimethyl-1,2-dihydroquinoline (dehydrodemethylethoxyquin (DHMEQ)) and through dimerization to form EQ dimer (EQDM) [39, 42]. On the other hand, in an oxidizing medium other molecules can be formed, for example, 2,6-dihydro-2,2,4-trimethyl-6-quinolone (QI) or nitroxide radical (6-ethoxy-2,2,4-trimethyl-1,2-dihydroquinolin-N-oxyl) which is also a strong antioxidant [39, 42].

 Products of EQ oxidation were detected by different authors in fish oil and meal [22, 43–45]. According to He and Ackman [46] the following oxidization products of EQ dominate in fish meal and fish feed: 2,6-dihydro-2,2,4-trimethyl-6-quinolone (QI) and 1,8′-di(1,2-dihydro-6-ethoxy-2,2,4-trimethylquinoline) (EQDM). At high storage temperature neither QI nor EQDM accumulates; however, another product of EQ oxidation, 2,4-dimethyl-6-ethoxyquinoline, is stable [41]. As it was pointed out earlier, ethoxyquin oxidization products also possess antioxidative properties. The EQDM and QI show 69% and 80% of EQ efficacy, respectively (studies on fish meal) [12]. On the other hand, in the studies of Thorisson et al. [45] quinone imine (QI) and EQ nitroxide were also powerful antioxidants, while EQDM, the main product of EQ oxidation, showed little or no antioxidant behavior. 

 Antioxidant activity of EQ was also demonstrated in experiments performed both *in vivo *and *in vitro*. Antimutagenic effect of this antioxidant was observed in mice, rats, and Chinese hamsters treated with cyclophosphamide, an agent widely used in cancer chemotherapy [47–49]. During cyclophosphamide bioactivation reactive oxygen species are formed which can cause damage of genetic material [50, 51]. EQ reduced the number of chromosome aberrations, micronuclei, and dominant lethal mutations induced by the anticancer drug [47–49]. There were also some reports that EQ can modify carcinogenic response to different carcinogens [35, 52, 53]. EQ given to Fischer 344 rats in diet completely prevented the formation of aflatoxin B1-induced preneoplastic liver lesions [52, 53].

 In *in vitro* experiments with human lymphocytes, antioxidant activity of EQ was observed in the comet assay (the method used to detect single- and double-strand DNA breaks, cross-links, and alkaline labile sites) and in micronucleus test (the method for the detection of micronuclei induced by clastogens or aneugens). EQ used at the concentrations ranging from 1 *μ*M to 10 *μ*M protected human lymphocytes against DNA damage caused by hydrogen peroxide (H_2_O_2_, 10 *μ*M) [17]. This antioxidant also reduced the number of micronuclei caused by H_2_O_2_ used at concentration of 75 *μ*M. However, the significant reduction was evident only in the case of lower EQ concentrations (5 *μ*M, 10 *μ*M) with no effect at higher concentration [17].

## 5. Adverse Effects of EQ: *In Vivo* and *In Vitro* Studies

 Different phenolic antioxidants may be used in animal feed, such as BHA (butylated hydroxyanisole), BHT (butylated hydroxytoluene), and the most efficacious EQ. The levels of the antioxidants in finished feed should not be higher than 150 ppm for EQ and 200 ppm for BHT and BHA (U.S. Food and Drug Administration permissions). The fact that efficient antioxidants work optimally when they are used at low concentrations is their remarkable characteristic. On the other hand, when antioxidants are used at high concentrations they act as prooxidants. The impact of these compounds depends on their concentration as well as on other factors such as metal-reducing potential, chelating behaviour, solubility, and pH. The effect of antioxidants on living organisms also depends on their bioavailability and stability in tissues [54, 55]. Phenolic antioxidants under favorable conditions may be converted to phenoxyl radicals with prooxidant activity [55]. It was shown that dissolved EQ may exist partly in the free radical form which it was also detected in the compound itself [56]. Therefore, ethoxyquin nitroxide which is produced by EQ oxidation similarly as other nitroxide molecules (e.g., tempol) may also show prooxidative properties [57]. Formation of free oxygen species as a result of using too high EQ concentrations can cause adverse health effects in animals fed with EQ containing feed or in people consuming meat from farmed animals, for example, different fishes.

The studies on EQ prooxidant activity and toxicity associated with it were performed both *in vivo* and *in vitro*. Dogs are most susceptible to the harmful effects of EQ, and first reports of such effects were received by FDA in 1988. The symptoms observed by dog owners and veterinarians were liver, kidney, thyroid and reproductive dysfunction, teratogenic and carcinogenic effects, allergic reactions, and a host of skin and hair abnormalities [7]. According to the studies on dogs and laboratory animals it was shown that ethoxyquin had little acute toxicity, except when it is administered parenterally. Values of LD_50_ for EQ are 1700 mg kg^−1^ bw (rats, oral gavage), >2000 mg kg^−1^ bw (rats, dermal treatment, 24 h), ~900 mg kg^−1^ bw (mice, intraperitoneal administration), and ~180 mg kg^−1^ bw (mice, intravenous administration) [13]. Despite species differences in the majority of animals treated with EQ at the concentrations higher than those permitted in animal feed, the same characteristic symptoms and pathologies appeared such as weight loss, liver, and kidney damage, alterations of alimentary duct (Table 4). The concentration of 100 ppm (equivalent to 2.5 mg kg^−1^ bw per day) was considered to be a minimal-effect level for clinical signs of toxicity and liver effects in dogs, the most susceptible animals [13, 14].

 Detrimental effects of EQ were also seen when the experiments were performed at the cell metabolism level. Hernandez et al. [64] and Reyes et al. [65] analysed the impact of EQ on the metabolic pathways of rat renal and hepatic cells, as well as on mitochondria and submitochondrial particles obtained from bovine heart and kidney. They observed influence of EQ on energy processes in cells. EQ inhibited renal Na^+^, K^+^-ATPase activity involved in ion transport [64]. The authors suggested that EQ interacted with site I of the mitochondrial respiratory chain, and it resulted in inhibition of oxygen consumption in the mitochondria of kidney and liver cells when glucose was a respiratory substrate. The effect was dose dependent. 

 More than 30 years ago when EQ began to be more commonly used in animal feed research started to assess its mutagenicity with the use of Ames test which is performed on different *Salmonella typhimurium* strains. The results were equivocal as some results were negative [66–68], but the positive effects were also observed [69, 70]. It was also shown that EQ enhanced the mutagenic activity of DMBA (3,2′-dimethyl-4aminobiphenyl), a compound having carcinogenic properties [70]. Ethoxyquin was reported to both enhance and inhibit genetic changes induced by known carcinogens; on the other hand it can also lead to cancer in exposed animals. Manson et al. [53] observed in Fischer 344 rats that EQ caused severe damage in kidney. Many hyperplastic and putative preneoplastic tubules were found which suggested that EQ may be exerting a carcinogenic effect. Similar effects were observed earlier by Ito et al. [59] in relation not only to the kidney but also to the urinary bladder.

 Possible carcinogenicity of EQ is probably connected with its prooxidant activity and induction of reactive oxygen radicals which cause DNA damage. DNA damage is usually repaired by cellular repair system, but if it is severe or there are too many lesions, this leads to programmed cell death (apoptosis). Sometimes, however, the programmed cell death pathway is damaged so when the defense mechanisms fail there is no way to stop a cell from becoming a cancer cell. Some *in vitro* studies showed both cytotoxic effects of EQ leading to cell apoptosis or necrosis and damage of genetic material at DNA or chromosome levels. Cytotoxic effects of pure EQ (purity > 97%) were studied *in vitro *with the use of human lymphocytes. The IC_50_ value (the concentration causing 50% growth inhibition) for EQ determined after 72-hour treatment of the cells in the MTT assay was 0.09 mM [71]. This antioxidant significantly reduced viability of lymphocytes detected with trypan blue exclusion method after 24-hour treatment at the concentrations of 0.25 and 0.5 mM (cell divisions were stimulated by phytohemagglutinin, (PHA)) [19] or of 0.05 mM and higher when 1-hour treatment was performed [72]. EQ-induced apoptosis by observed in *in vitro *cultured human lymphocytes starting from 0.05 mM concentration and the detected number of apoptotic cells depended on the treatment time [71]. Ethoxyquin caused also DNA damage in the comet assay [72] however, most lesions could be repaired by cellular DNA repair systems [73]. On the other hand, the results obtained with the use of chromosome aberration test showed that unrepaired DNA damage induced by EQ could lead to permanent changes in genetic material [16, 74]. Błaszczyk et al. [16] and Gille et al. [74] showed that this antioxidant induced chromosome aberrations such as breaks, dicentrics, atypical translocated chromosomes, or chromatid exchanges in human lymphocytes and Chinese hamster ovary cells. These aberrations are known to have serious biological consequences [75]. 

## 6. Analogues and Derivatives of EQ

 Because of adverse health effects caused by EQ it is reasonable to search for new antioxidants as effective in scavenging free radicals as EQ which produce no such problems. In the paper of de Koning [12] nine analogues of EQ prepared to compare their antioxidant efficacy with that of the parent chemical are presented. The compounds have been tested in a refined fish oil and subsequently some of the most promising ones have been also tested in fish meal. It was noted that the results obtained in fish oil were not always the same as in fish meal, for example, hydroxyquin (1,2-dihydro-6-hydroxy-2,2,4-trimethylquinoline; Figure 1) was 3.5 times as effective as EQ in fish oil, while only 3/4 of its efficacy was observed in fish meal. In the case of another compound—hydroquin (1,2-dihydro-2,2,4-trimethylquinoline; Figure 1) antioxidant efficacy in relation to EQ was 101% in fish oil and 52% in fish meal. Despite the lower efficiency of this compound in fish meal the author stated that hydroquin can compete with EQ as an antioxidant of choice [12]. The reason is that preparation of hydroquin based on aniline and acetone is more cost-effective than that of EQ whose production requires p-phenetidine (more expensive than aniline). Hydroquin was earlier patented as an antioxidant in animal feeds in 1997 [76]. The 2-year dermal research with the use of F344/N rats and B6C3F1 mice conducted under the National Toxicology Program [77] showed that the compound was not carcinogenic, but the studies performed by Sitarek and Sapota [78] showed its teratogenic properties. 

 In 2000 Dorey et al. [15] presented the report concerning the synthesis and biological properties of a new class of antioxidants based on the EQ backbone. The studies were performed to search for new quinolinic derivatives with radical scavenging activity, potential candidates for central nervous system protection. EQ is not suitable for that as it has been shown to exhibit significant hypothermic effect, probably as a result of an inhibition of electron transport in the mitochondrial respiratory chain [65]. Dorey et al. [15] synthesized and studied many 1,2-dihydro and 1,2,3,4-tetrahydroquinolines and then selected for further evaluation a group of antioxidants (5 compounds) with high radical scavenging capacities, relatively low toxicity, and moderate hypothermia. The compounds belonging to the group of 1,2,3,4-tetrahydroquinolines (e.g., 6-ethoxy-2,2,5,7-tetramethyl-1,2,3,4-tetrahydroquinoline, characterized with the lowest toxicity and high radical scavenger capacity) are structurally similar to 2,2,4,7-tetramethyl-1,2,3,4-tetrahydroquinoline synthesized and tested in our laboratory (Figure 1) [17]. The latter compound also had promising features: its antioxidant activity was comparable to that of EQ, but its cytotoxicity and genotoxicity studied with the use of human lymphocytes *in vitro* were significantly lower. We believe that this chemical is worth of detailed studies to confirm its usefulness as a food preservative. Some other EQ derivatives and salts were also studied for cytotoxicity, genotoxicity, and antioxidant activity, namely, the complexes of ethoxyquin with flavonoids (rutin or quercetin), ethoxyquin hydrochloride, ethoxyquin phosphate, ethoxyquin L-ascorbate, ethoxyquin *n*-hexanoate, ethoxyquin salicylate, and ethoxyquin salt of Trolox C [19, 79–82]. The biological properties of the compounds were analysed with the use of MTT, TUNEL, and trypan blue staining methods (cytotoxicity testing), comet assay (genotoxicity testing), and micronucleus test (mutagenicity testing). From among the compounds tested ethoxyquin phosphate (EQ-F) was the least toxic (Table 5)—its cytotoxic and genotoxic activities in comparison with those of EQ were reduced positively (IC_50_ = 0.8 mM versus 0.09 mM for EQ) [71]. On the other hand, antioxidant activity of EQ-F was observed, but it was the lowest of the tested compounds [82]. The studies showed that all the tested compounds were less toxic to human lymphocytes than EQ, and the antioxidant activity of four of them (ethoxyquin *n*-hexanoate, ethoxyquin complex with quercetin, ethoxyquin L-ascorbate, and ethoxyquin salicylate) was comparable with that of EQ [79–82]. The results obtained indicate that their use as antioxidants may be considered.

## 7. Food Safety Aspect

EQ safety has been under consideration for many years. The level of this antioxidant in animal feeds should not be higher than 150 ppm (U.S. Food and Drug Administration permissions). The approved uses of ethoxyquin in animal feeds are addressed in the Code of Federal Regulations (CFR), Title 21, Parts 573.380 and 573.400, and established tolerances are in Part 172.140. On the one hand, the observed adverse health effects (firstly in dogs) could be caused by the fact that the animals ate a lot of feed containing EQ, but on the other hand, it could also be the result of its excessive amounts in the feed. Ethoxyquin is added to animal feed either directly or indirectly as a component of an ingredient. From time to time FDA reminds industry about labeling and safe use requirements for ethoxyquin, but if it is added at the ingredient level this is not always indicated. 

 Another important safety issue is the presence of EQ oxidation and EQ metabolism products in animal feed or in foods prepared from farmed animal meat. de Koning [12] described main products of EQ oxidation which can be observed in stored feeds or in fish meal: EQ dimer (EQDM, 1,8′-di(1,2-dihydro-6-ethoxy-2,2,4-trimethylquinoline) and quinone imine (QI, 2,6-dihydro-2,2,4-trimethyl-6-quinolone). Both compounds were shown to be potent antioxidants, but they can also have detrimental effect, especially so because the half-life of the dimer was considerably greater than that of EQ [26, 28]. In the recent studies no adverse toxicological effects of EQDM, in terms of kidney and liver function, were observed in *in vivo *experiments with F344 rats exposed for 90 days to the compound [37]. On the other hand, Augustyniak et al. [83] showed that EQDM, similarly as EQ, was cytotoxic and genotoxic to human lymphocytes. Toxicity of QI has not been studied yet, but the results obtained by the authors indirectly indicated that the compound could be cytotoxic to human cells. 

 The levels of the parent compound (EQ) in meat of farmed animals are usually lower than MRL (Maximum Residue Level) [20, 84], but EQ oxidation products are usually not controlled. It was shown that EQDM and other EQ residues can be present in different animal tissues [23, 24, 26, 28, 37]. In the studies of Bohne et al. [23] in which Atlantic salmons were fed for 12 weeks with the feed containing this antioxidant, four compounds were identified in their muscles: parent EQ (6-ethoxy-1,2-dihydro-2,2,4-trimethylquinoline), deethylated EQ (6-hydroxy-2,2,4-trimethyl-1,2-dihydroquinoline), quinone imine (2,6-dihydro-2,2,4-trimethyl-6-quinolone, QI), and EQ dimer (1,8′-di(1,2-dihydro-6-ethoxy-2,2,4-trimethylquinoline, EQDM). It was also shown that the concentration of EQ in fish muscle was proportional to the duration of exposure and the level of EQ in the feed [23]. The same linear increase was seen for EQDM, the main metabolite of EQ, and the sum of EQ and EQDM. Bohne et al. [23] found that the level of EQ and its metabolites in fish muscle could be predicted from the level of dietary EQ and then controlled, but because it is not the only factor which may affect the levels of EQ and its metabolites in the salmon tissue (others for example are fish size and age), the concentration of EQ and EQDM in fish ready for consumption may be higher than that observed in their studies. In their experiments it was shown that the elimination of EQ from salmon was concurrent with significant increase in the level of EQDM, and they concluded that mandatory 14 days of depuration were not sufficient for elimination of EQ residues—it is mainly because EQDM is characterized by the considerably longer half-life than that of EQ. Moreover, EQDM accounted for 99% of the sum of the two compounds (EQ and EQDM), and its toxicological effects in animals and humans are unknown. EQ and EQ dimer were also detected in similar amounts not only in Atlantic salmon, but also in other commercially important species of farmed fishes (halibut, rainbow trout) by Lundebye et al. [24]. They found that in Atlantic salmon, halibut, and rainbow trout the concentration of EQDM was more than 10-fold higher than that of EQ. The authors estimated that consumer exposure to EQ from a single portion (300 g) of skinned-fillets of different species of farmed fish could amount up to 15% of the ADI. In the light of data concerning the presence of EQDM in the body of farmed fishesand providing that EQ dimer was included in the ADI, the EQ and EQDM intake from a single portion of Atlantic salmon would be close to ADI [24]. Farmed fish is probably the major source of EQ and its residues for European consumers (its use as a food additive is forbidden). In our opinion, however, both fish and other farmed animals, for example, chickens, should be controlled for the presence of not only EQ, but also EQDM, its main oxidation product.

## 8. Conclusion

Ethoxyquin has been used as an antioxidant in animal feed for several decades and despite the search for new compounds that could be used as free radical scavengers, it is still the most effective antioxidant. The negative health effects in domestic animals fed with EQ containing feed were observed some years ago, but the presence of its approved doses should not be hazardous. Toxicity and mutagenicity of EQ were observed in *in vivo* and *in vitro *studies showing its potential harmful effects. This makes it very important to label all products and ingredients to which EQ is added and to comply with the recommended doses. Additionally, the results of the studies on products of EQ oxidation, especially EQDM, detected in farmed animal tissues indicate that it should be under control and some regulations should be introduced.

## Figures and Tables

**Table 1:** Permitted amounts of EQ in different products approved by FDA∗.

Product	Dose [ppm]
Animal feed	150
Spices	150
Uncooked fat of meat from animals (except poultry)	5
Uncooked liver and fat of poultry	3
Uncooked muscle meat of animals	0.5
Poultry eggs	0.5
Milk	0
Pear	3

^*^According to the Code of Federal Regulations (CFR), Title 21, Parts 573.380, 573.400, 172.140.

**Table 2:** Physical and chemical properties of ethoxyquin.

Properties of EQ	
Molecular formula	C_14_H_19_NO
Molecular mass	217.34 [g mol^−1^]
IUPAC name	1,2-dihydro-2,2,4-trimethylquinolin-6-yl ethyl ether
CAS name	6-ethoxy-1,2-dihydro-2,2,4-trimethylquinoline
Chemical class	Quinoline
Melting point	0°C^a^
Boiling point	123–125°C at 2 mm Hg^a^
Solubility	Insoluble in water, soluble in fat and animal and plant oils, and soluble in ethanol, methanol, DMSO, and DMF
Vapor density	7.48 (AIR = 1)^a^
Stability	Stable under ordinary conditions, storage temp. 0–6°C
Spectral properties	Index of refraction: *n*_*D*_ 1.569–1.672 at 25°C^a^
	Max absorption: 354 nm. Intense mass spectral peaks: 202 *m/z* (100%), 108 *m/z* (53%), 174 *m/z* (48%), and 137 *m/z* (36%)^a^
	^ 13^C NMR in CDCl_3_ EQ (8% solution) *δ*_*C*_ in ppm: 151.2 (EQ-C-6); 137.6 (EQ-C-4); 129.5 (EQ-C-3); 128.5 (EQ-C-9); 122.8 (EQ-C-10); 114.6 (EQ-C-8); 113.6 (EQ-C-7); 111.1 (EQ-C-5); 64.2 (EQ-CH_2_-O); 51.6 (EQ-C-2); 30.4 (EQ-CH_3_-4); 18.5 (EQ-CH_3_-4); 15.0 (EQ-CH_3_-CH_2_-O-)^b^

^a^According to the National Library of Medicine HSDB Database (last revision date: 2003) [18].

^b^According to Błaszczyk and Skolimowski [19].

DMSO: dimethyl sulfoxide, DMF: *N,N*-dimethylformamide.

**Table 3:** Metabolite/degradation products of EQ detected in different organisms.

	EQ metabolite/degradation product	Organism
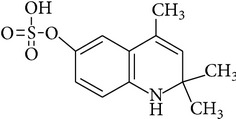	1,2-Dihydro-6-hydroxy-2,2,4-trimethylquinoline sulphate	Rats^1^
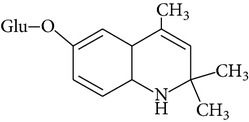	1,2-Dihydro-6-hydroxy-2,2,4-trimethylquinoline glucuronide	Mice^1^
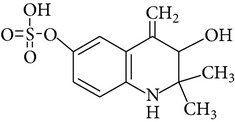	1,2,3,4-Tetrahydro-3,6-dihydroxy-4-metylene-2,2-dimethylquinoline sulphate	Rats^1^
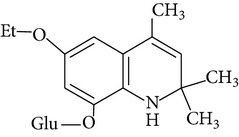	1,2-Dihydro-6-ethoxy-8-hydroxy-2,2,4-trimethylquinoline glucuronide	Rats^1^ Mice^1^
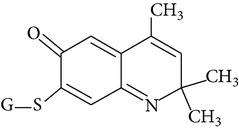	8-(S-glutathionyl)-2,2,4-trimethylquinol-6-one	Rats^1^ Mice^1^
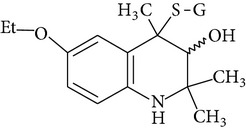	1,2,3,4-Tetrahydro-6-ethoxy-3-hydroxy-4-(S-glutathionyl)-2,2,4-trimethylquinoline	Rats^1^ Mice^1^
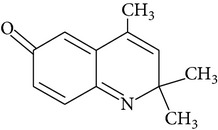	2,6-Dihydro-2,2,4-trimethyl-6-quinolone (quinone imine (QI))	Rats^1^ Atlantic salmon^2^
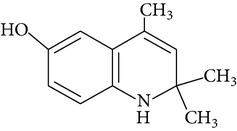	6-Hydroxy-2,2,4-trimethyl-1,2-dihydroquinoline (deethylated EQ (DEQ))	Atlantic salmon^2^
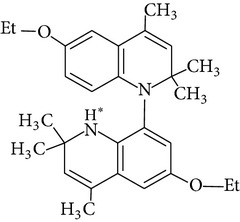	1,8′-Di(1,2-dihydro-6-ethoxy-2,2,4-trimethylquinoline) or 6,6′-diethoxy-2,2,2′,2′,4,4′-hexamethyl-1′,2′-dihydro-2*H*-1,8′-biquinoline (ethoxyquin dimer (EQDM)) ∗Instead of the hydrogen atom there is a methyl group, it gives methyl C-N ethoxyquin dimer, 6,6′-Diethoxy-1′,2,2,2′,2′,4,4′-heptamethyl-1′,2′-dihydro-2*H*-1,8′-biquinoline	Atlantic salmon^2^ Pears^3,4^
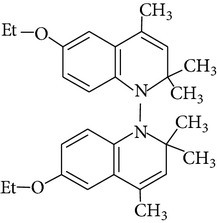	N-N Ethoxyquin dimer, 1,1′-di(1,2-dihydro-6-ethoxy-2,2,4-trimethylquinoline) or 6,6′-diethoxy-2,2,2′,2′,4,4′-hexamethyl-2*H,*2′*H-*1,1′-biquinoline	Pears^3,4^
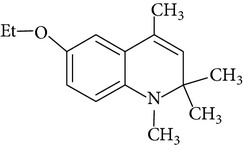	6-Ethoxy-1,2,2,4-tetramethyl-1,2-dihydroquinoline, methylethoxyquin (MEQ)	Pears^3,4^
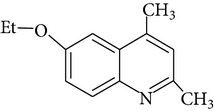	6-Ethoxy-2,4-dimethylquinoline, dehydrodemethylethoxyquin (DHMEQ)	Pears^3,4^
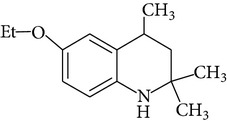	6-Ethoxy-2,2,4-trimethyl-1,2,3,4-tetrahydroquinoline, dihydroethoxyquin (DHEQ)	Pears^3,4^
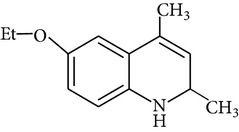	6-Ethoxy-2,4-dimethyl-1,2-dihydroquinoline, demethylethoxyquin (DMEQ)	Pears^3,4^

Glu: glucuronide, G: glutathione, Et: ethyl group (C_2_H_5_).

^1^Burka et al. [21], ^2^Berdikova Bohne et al. [26], ^3^Gupta and Boobis [14], ^4^JMPR [25].

**Table 4:** Harmful effects of EQ observed after its oral administration in different animals or in humans (contact exposure).

The harmful effect	Animals
Loss of weight	Marmosets^1^, rats^2^, dogs^2^, mice^2^, rabbits^3^
Changes in liver	Marmosets^1^, rats^1,4^, dogs^2^, mice^2^, broiler chickens^2^, tilapia^5^
Changes in kidney	Marmosets^1^, rats^2,6,7^, dogs^2^, broiler chickens^2,8^
Changes in alimentary duct	Marmosets^1^, dogs^2^, mice^2^, broiler chickens^2,8^
Changes in urinary bladder	Rats^2^
Anemia	Marmosets^1^
Changes in mitochondria	Rats^2^
Lethargy	Rabbits^3^
Colored urine, skin, or fur	Dogs^2^, rats^2^
Increase in mortality	Broiler chickens^2,8^
Effect on immunity	Tilapia^5^
Condition factor: the final body weight in relation to body length of fish	Large yellow croaker^9^
Allergy (contact exposure)	Humans^10,11,12^

^1^McIntosh et al. [58], ^2^Drewhurst [13], ^3^Little [10],^4^Ito et al. [59], ^5^Yamashita et al. [60], ^6^Neal et al. [61], ^7^Manson et al. [53], ^8^Leong and Brown [62], ^9^Wang et al. [63], ^10^Rodríguez-Trabado et al. [9], ^11^Brandao [5], ^12^Savini et al. [6].

**Table 5:** Comparison of different activities of ethoxyquin and its derivatives based on the data presented by Błaszczyk and Skolimowski [19, 71, 80–82] and Błaszczyk et al. [79].

Activitity	Activity from the lowest (left) to the highest (right) one
Cytotoxicity	EQ-F < EQ-HCl < EQ-C < EQ-S < EQ-T < EQ-Q < EQ-R < EQ-H < EQ
Genotoxicity	EQ-F < EQ-C < EQ-H < EQ-HCl < EQ-Q < EQ-T < EQ-S < EQ-R < EQ
Antioxidant activity	EQ-F < EQ-HCl < EQ-R < EQ-T < EQ-S < EQ-C < EQ-Q < EQ < EQ-H

EQ-F: ethoxyquin phosphate; EQ-HCl: ethoxyquin hydrochloride; EQ-C: ethoxyquin L-ascorbate; EQ-S: ethoxyquin salicylate; EQ-T: ethoxyquin salt of Trolox C; EQ-Q: ethoxyquin complex with quercetin; EQ-R: ethoxyquin complex with rutin; EQ-H: ethoxyquin *n*-hexanoate; EQ: ethoxyquin.
